# Review of the genus *Roeslerstammia*, with a new species from China (Lepidoptera, Roeslerstammiidae)

**DOI:** 10.3897/zookeys.668.11896

**Published:** 2017-04-13

**Authors:** Toshiya Hirowatari, Guo-Hua Huang, Min Wang

**Affiliations:** 1 Entomological Laboratory, Faculty of Agriculture, Kyushu University, 6–10–1 Hakozaki, Fukuoka, 812–8581 Japan; 2 Hunan Provincial Key Laboratory for Biology and Control of Plant Diseases and Insect Pests, Hunan Agricultural University, Changsha 410128, Hunan, China; 3 Department of Entomology, South China Agricultural University, Guangzhou 510640, Guangdong, China

**Keywords:** Genitalia, Hunan, India, morphology, new species, taxonomy

## Abstract

The new species *Roeslerstammia tianpingshana* **sp. n.** is described from Hunan, China as the first record of the genus in the country. Examination of two enigmatic Indian species, *R. metaplastica* Meyrick, 1921 and *R. hemiadelpha* Meyrick, 1922, revealed that the latter is a synonym of the former. The male and female genitalia of *R. metaplastica* are described and illustrated for the first time. A checklist for the genus is given.

## Introduction

The family Roeslerstammiidae (= Amphitheridae) includes 57 species, mostly Australian, with one genus (*Roeslerstammia* Zeller, 1839) extending from Europe to Japan, and several genera (e.g., *Agriothera* Meyrick, 1907 and *Telethera* Meyrick, 1913) in the Oriental tropics (Heppner 2008). Two species of *Agriothera* were recorded from mainland China (Huang et al. 2008). The genus *Roeslerstammia* is widespread through the Palaearctic region and Kyrki (1983) indicated seven species in a tentative checklist. However, three species have been synonymized, so that currently only four species, *R. erxlebella* (Fabricius, 1787) and *R. pronubella* ([Denis & Schiffermüller], 1775) from Europe to Japan, and *R. metaplastica* Meyrick, 1921 and *R. hemiadelpha* Meyrick, 1922 from India are included in the genus (Heppner 2005). Until now no species of the genus *Roeslerstammia* have been recorded from China.

In May, 2009, an unknown species of *Roeslerstammia* was collected at Tianpingshan, in the northern part of Hunan Province, China. As a result of examination of morphological characters such as the wing markings, wing venation, and genitalia, it was concluded that it was a new species of *Roeslerstammia*, and it is described here. In addition, two enigmatic species of *Roeslerstammia* from India (Punjab) described by Meyrick, which have never been examined since the original descriptions, are also investigated in order to clarify their identities on the basis of examination of the genitalia.

## Materials and methods

Field surveys (light trap) were conducted in Tianpingshan (1,500 m), Badagongshan National Nature Reserve, Hunan Province, China on May 26–27, 2009 and on August 12–14, 2014. As for the two Indian species of the genus *Roeslerstammia*, syntype specimens deposited in the collection of the Natural History Museum (BMNH) were examined. Interocular index (= vertical eye diameter/inteocular distance) of Davis (1975) was calculated for description of eye size. Wing venations were examined after scales were removed and wings were stained with acetocarmine and embedded in Canada balsam on slide. Male and female genitalia were examined after the abdomen was macerated for about 5 minutes in 10% KOH heated in a boiling water bath. SEM photographs of the head were taken using HITACHI SU1510.

### Abbreviations

**BMNH** The Natural History Museum, London.

**ELKU** Entomological laboratory, Faculty of Agriculture, Kyushu University, Fukuoka.

**HUNAU** Hunan Agricultural University, Changsha.

## Taxonomy

### Roeslerstammia metaplastica

Taxon classificationAnimaliaLepidopteraRoeslerstammiidae

Meyrick

Figs 1 , 2 , 3

Roeslerstammia metaplastica Meyrick, 1921: 439, lectotype here designated; Kyrki 1983: 322; Heppner 2005: 27.
Roeslerstammia hemiadelpha Meyrick, 1922: 553, lectotype here designated; Kyrki 1983: 322. **syn. n.**
Roeslerstammia hemidelpha (!): Heppner 2005: 26. Misspelling.

#### Type material.

**Figure 1.**
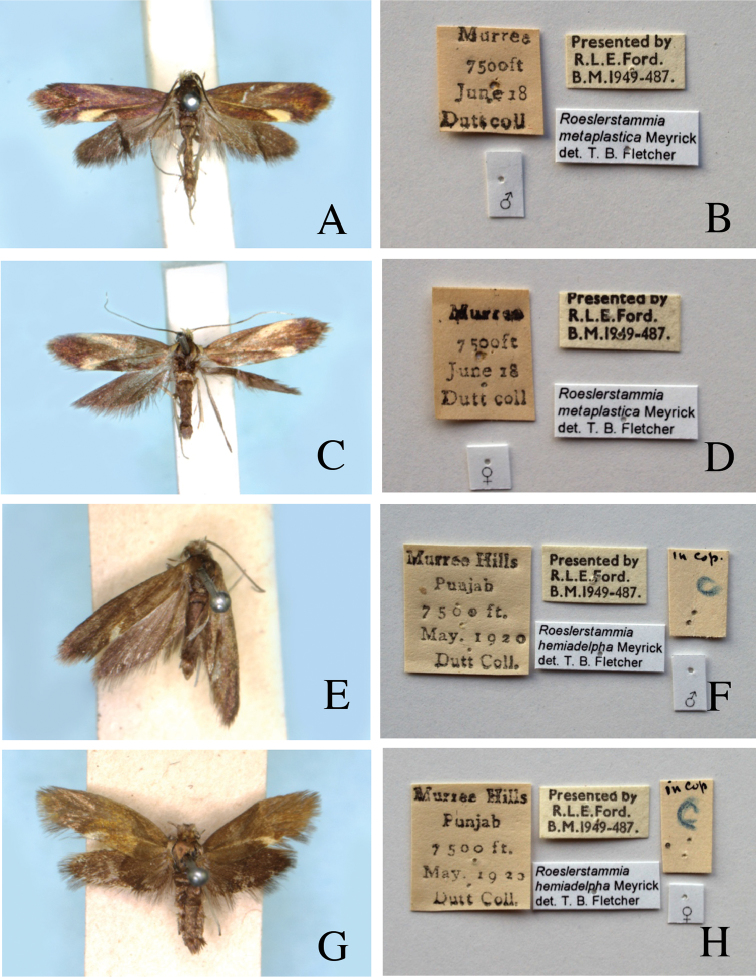
Type series of *Roeslerstammia* spp. from India. **A** Lectotype of *R. metaplastica* Meyrick, 1921, male **B** *Ditto*, labels **C** Paralectotype of *R. metaplastica* Meyrick, 1921, female **D** *Ditto*, labels **E** Lectotype of *R. hemidelpha* Meyrick, 1922, male **F** *Ditto*, labels **G** Paralectotype of *R. hemidelpha* Meyrick, 1922, female **H** *Ditto*, labels.

Lectotype ♂ (here designated), “Murree [Hills, Punjab] / 7500 ft/June 18 / Dutt Coll”, “Presented by / R.L.E.Ford. / B.M.1949–487.”, “*Roeslerstammia metaplastica* Meyrick / det.T.B.Flecher”, “♂” (Fig. 1A, B), in BMNH. B.M. Genitalia slide No. 29548.

Paralectotype ♀, same labels as lectotype except for “♀” (Fig. 1C, D) , in BMNH. B.M. Genitalia slide No. 29549.

Lectotype ♂of *Roeslerstammia hemiadelpha* Meyrick, 1922 (here designated), “Murree Hills / Punjab / 7500 ft / May 1920 / Dutt Coll”, “Presented by / R.L.E.Ford. / B.M.1949–487.”, “*Roeslerstammia hemiadelpha* Meyrick / det.T.B.Flecher”, “in Cop /C” “♂” (Fig. 1E, F) , in BMNH. B.M. Genitalia slide No. 29550.

Paralectotype ♀of *Roeslerstammia hemiadelpha* Meyrick, 1922, same labels as lectotype except for “♀” (Fig. 1G, H), in BMNH. B.M. Genitalia slide No. 29551.

#### Diagnosis.

Distinguished from the other species by the narrow triangular creamy-white tornal spot of the forewing. In the male genitalia, the uncus is rectangular, apically bilobed and broad; the valva has a short sacculus terminating in a blunt process; the phallus is short, sinuate, and tapered toward the apex. In the female genitalia, the ductus bursae is slender, nearly straight; the corpus bursae is long-ellipsoidal, with a strongly sclerotized sword-shaped signum.

#### Description.

***Male*** (Fig. 1A, E).

Forewing length 5.3 mm. Wing expanse 11.3 mm.

*Head* vertex, including between antennae, with raised pale yellow hairs; frons smooth, ochreous with golden luster, laterally pale yellow along eyes. Eyes moderate, interocular index *ca* 0.8. Antenna filiform, *ca* 0.9× as long as forewing; scales in flagellar segments near the middle of the antenna somewhat raised; scape pale yellow on basal half and dark brown on distal half; flagellum dark brown on basal 2/3, white on distal third, densely ciliate with sensory hairs ventrally. Labial palpus slightly upcurved, relatively long *ca* 2.2 × as long as horizontal eye diameter, 3^rd^ segment as long as 2^nd^, entirely smooth, 2^nd^ pale yellow, 3^rd^ pale yellow with dark brown laterally.

*Thorax* tegula pale yellow (scales partly removed); mesonotum dark brown with metallic luster. Fore- and midlegs pale yellow, tarsomeres brown distally; hindleg pale yellow. Forewing, lanceolate, dark brown with metallic blue or golden luster; a narrow indistinct oblique marking present at basal 2/3 near costa, a line along fold from base, terminating in a narrow triangular creamy-white tornal spot; fringe dark brown. Hindwing dark brown, darker near apex; fringe dark brown.

*Abdomen* pale brown with golden luster, terminally with pale yellow tufts.

**Figure 2.**
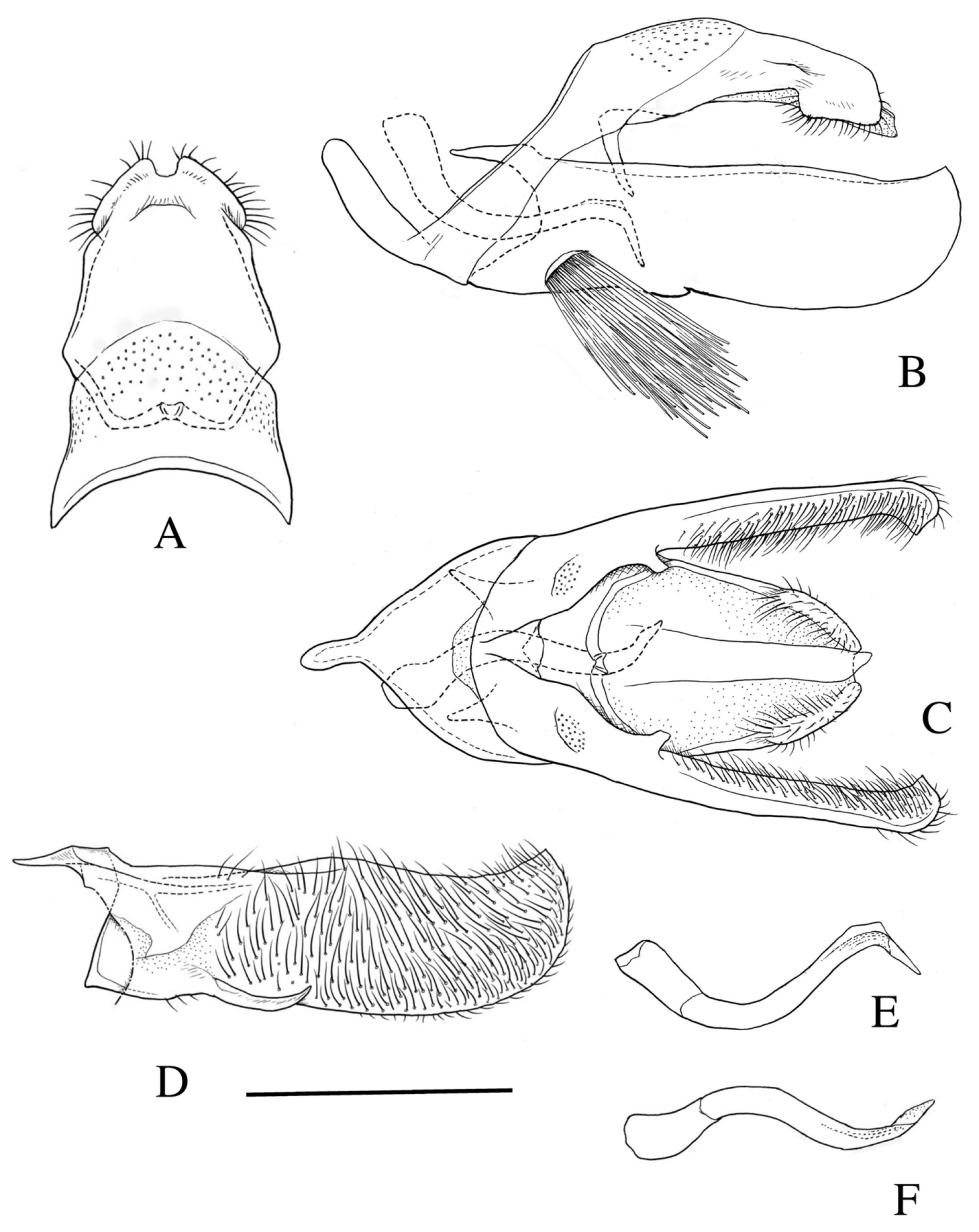
Male genitalia of *Roeslerstammia metaplastica* Meyrick, 1921, lectotype. **A** Dorsum (uncus and tegumen), dorsal view **B** Genitalia without phallus, lateral view **C** *Ditto* ventral view **D** Right valva, inner view **E** Phallus, lateral view **F** *Ditto*, dorsal view. Scale bar: 0.5 mm.

*Male genitalia* (Fig. 2). Uncus rectangular, apically broad and bilobed. Tegumen broad, slightly shorter than uncus. Gnathos consisting of two slender arms united medially with a membranous part. Valva rectangular; sacculus short, about 1/3 length of valva, terminating in a blunt process; a small pad of long hair scales near the base ventrally. Vinculum broad ventromedially; saccus cylindrical, as long as dorsal part of tegumen. Phallus, short, sinuate, tapered toward apex, with indistinct minute spine-like cornuti.

***Female*** (Fig. 1C, G).

Forewing length 5.5 mm. Wing expanse 12.1 mm.

Similar to male but differs as follows: scales in flagellar segments near the middle of the antenna not raised; flagellum without dense sensory hairs ventrally. Forewing with the oblique marking on costa broader, but indistinct in “*R. hemiadelpha*”.

**Figure 3.**
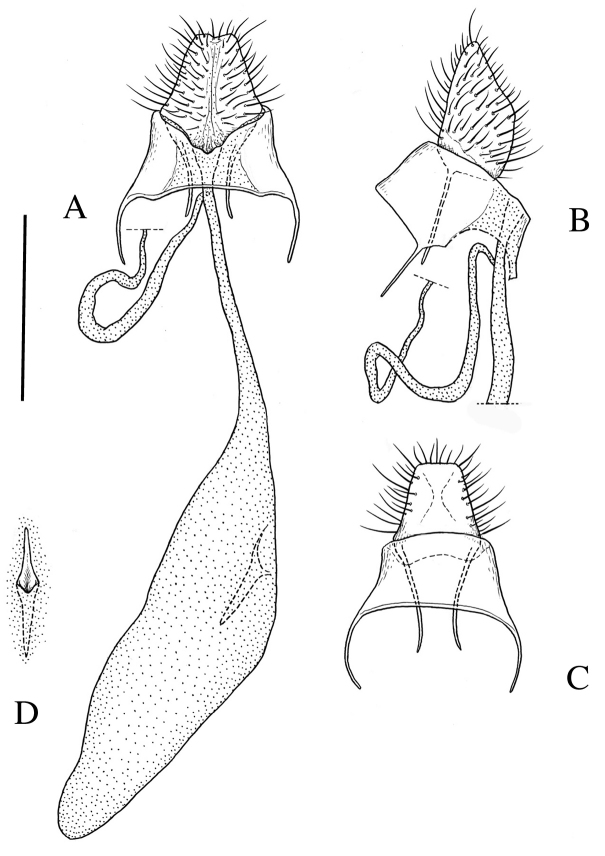
Female genitalia of *Roeslerstammia metaplastica* Meyrick, 1921, paralectotype. **A** Terminalia and bursa copulatrix, ventral view **B** Terminalia, lateral view **C** *Ditto*, dorsal view. **D** Signum. Scale bar: 0.5 mm.

*Female genitalia* (Fig. 3). Papillae anales broad and truncate in ventral view, nearly rectangular in lateral view. Apophysis posterioris slender, 0.7 × as long as papilla analis. Apophysis anterioris slender and moderate in length, 0.6 × as long as eighth tergite. Eighth tergite weakly sclerotized, dorsal posterior margin nearly straight. Ostium bursae situated at posterior margin of eighth abdominal segment, posterior margin weakly emarginate at middle. Ductus bursae slender, nearly straight. Ductus seminalis attached to ductus bursae near ostium. Corpus bursae long-ellipsoidal, with a strongly sclerotized sword-shaped signum.

#### Host plant.

Unknown.

#### Distribution.

India (Punjab).

#### Remarks.

Meyrick (1921) described *R. metaplastica* from the “Murree Hills, Punjab” based on five specimens collected by Dutt in June. Subsequently, Meyrick (1922) described *R. hemiadelpha* based on six specimens, which were collected by the same collector in the same locality in May 1920. Meyrick distinguished his two species on the basis of external characters such as coloration and wing markings (see Fig. 1). Since then, these specimens have not been studied again and they have been regarded as two distinct species following Meyrick. Both the male and female genitalia of some syntypes of *R. metaplastica* and *R. hemiadelpha* were examined, but there were no differences in the shape of male or female genitalia between the two taxa. Therefore, it was concluded that the latter is a junior synonym of the former. Of the original syntype series, only one male and female of each taxon was found in BMNH: both males are selected as lectotypes.

### Roeslerstammia tianpingshana sp. n.

Taxon classificationAnimaliaLepidopteraRoeslerstammiidae

http://zoobank.org/C03D3623-B273-49E8-ACDF-C23364922139

**Figure 4.**
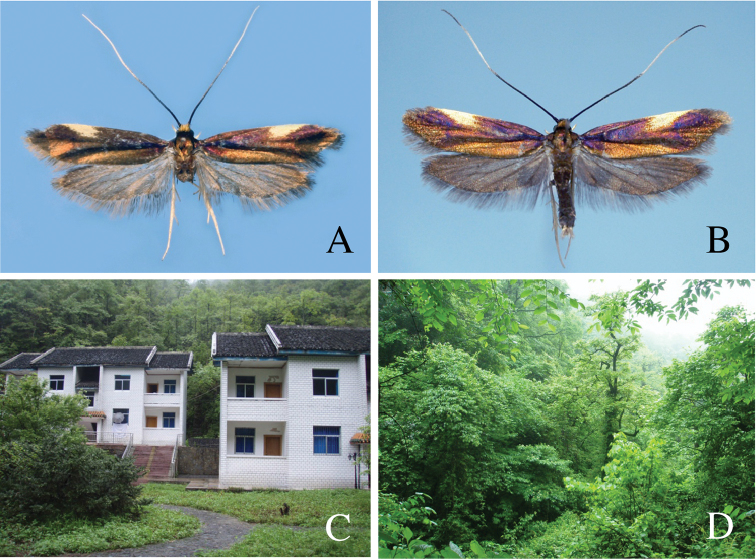
Adult and type locality of *Roeslerstammia tianpingshana* sp. n. **A** Holotype male **B** Paratype female **C** Type locality of *R. tianpingshana* sp. n., Tianpingshan, Hunan, China **D** Vegetation near the type locality.

**Figure 5.**
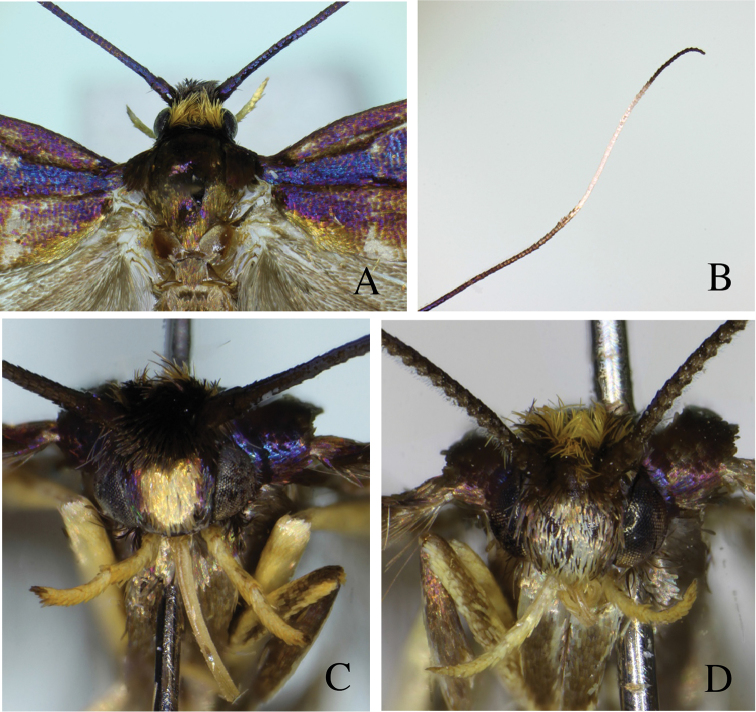
Head and thorax of *Roeslerstammia tianpingshana* sp. n. **A** Paratype female, dorsal view **B** *Ditto*, distal half of antenna **C** *Ditto*, head, frontal view **D** holotype, head, frontal view.

**Figure 6.**
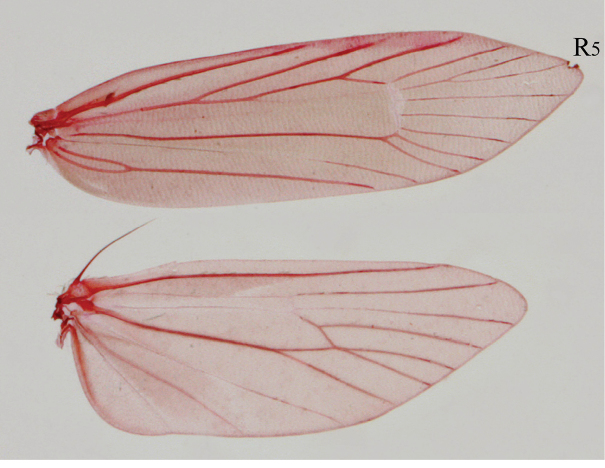
Wing venation of *Roeslerstammia tianpingshana* sp. n., male.

**Figure 7.**
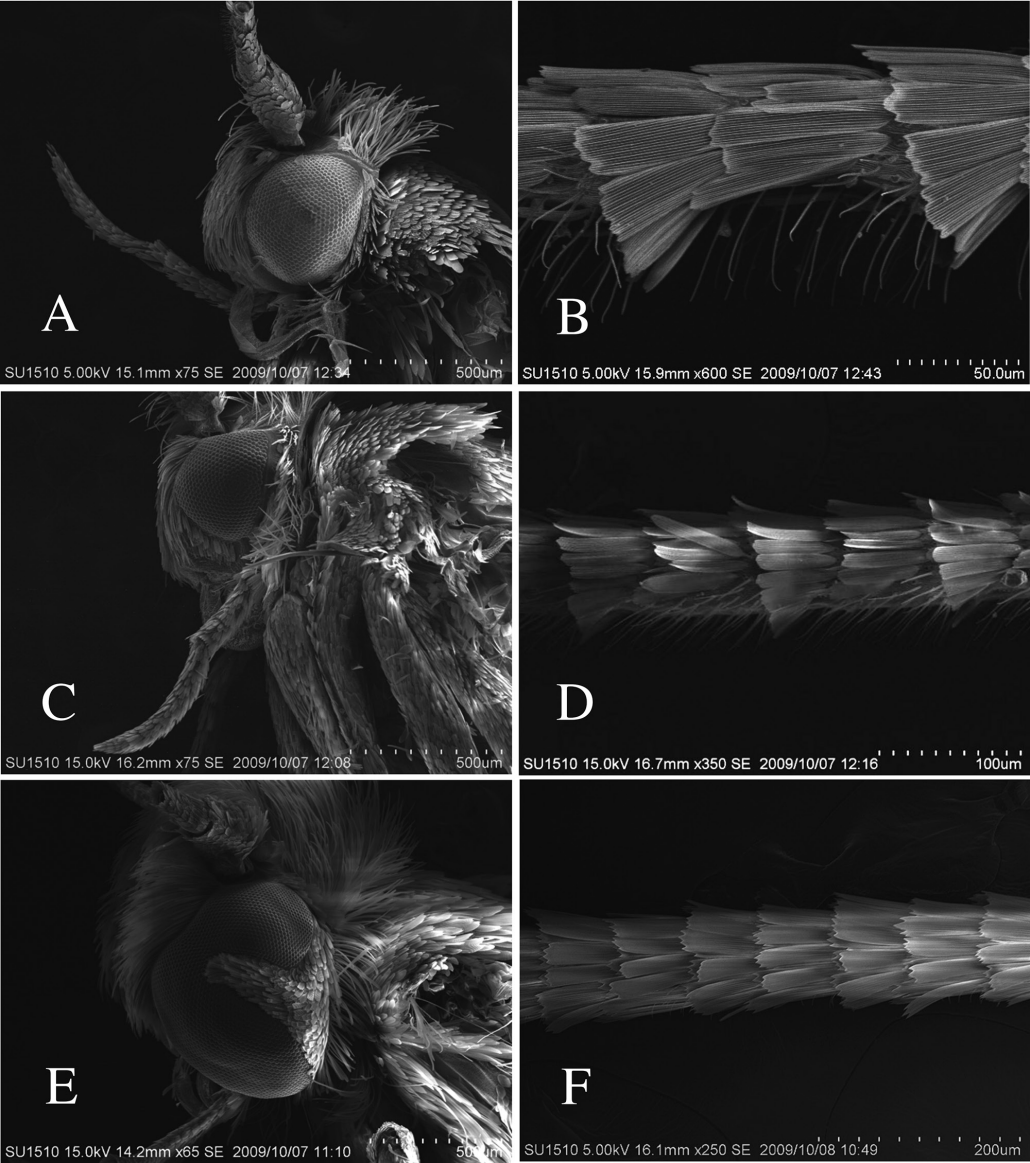
SEM images of head and antenna in Roeslerstammidae. **A–B** *Roeslerstammia tianpingshana* sp. n., male **C–D** *R. pronubella* male, Japan. **E–F** *Telethera blepharacma* Meyrick, 1913, male, Japan. **A, C, E** Lateral aspect of the head **B, D, F** Antenna, lateral aspects.

**Figure 8.**
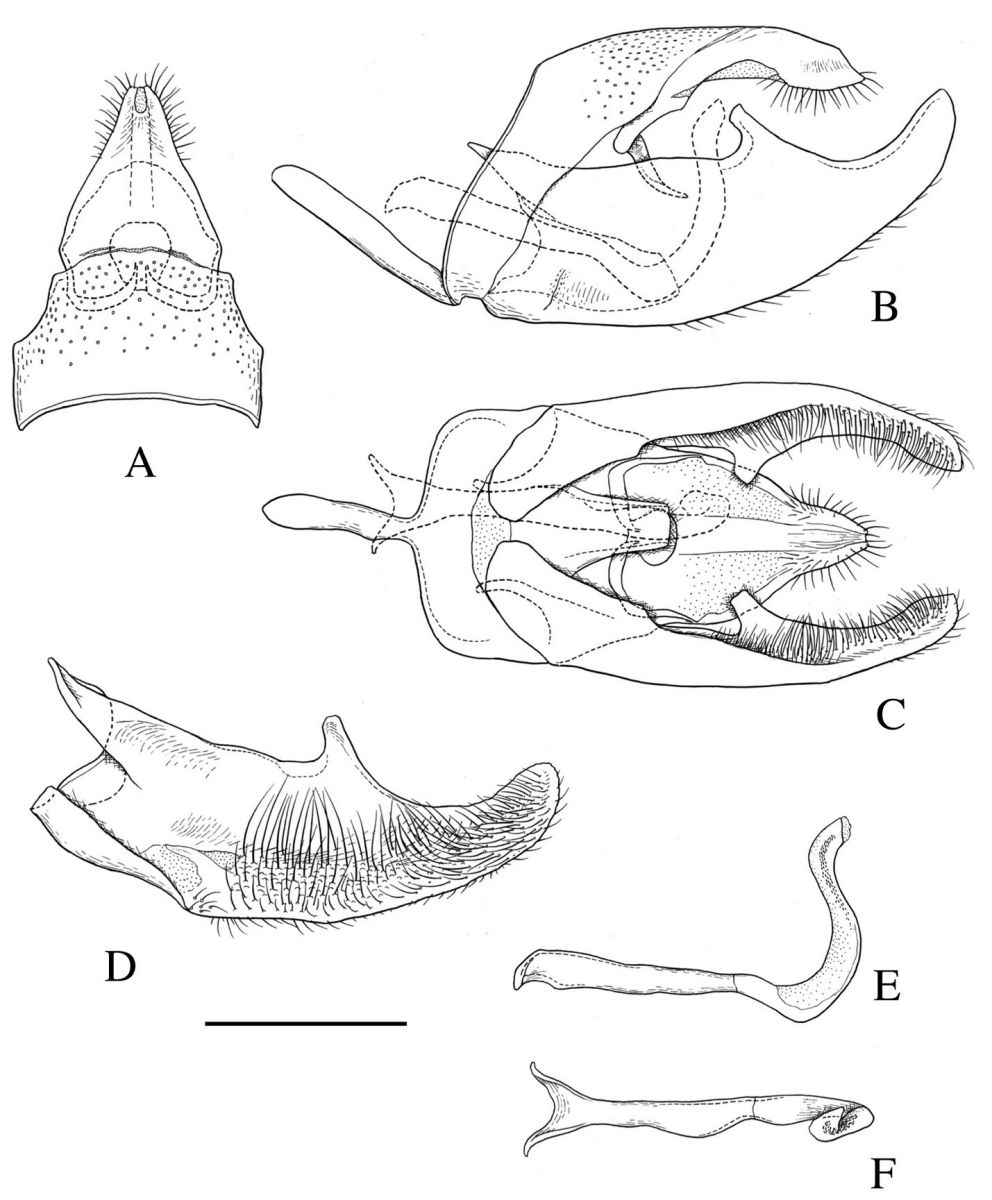
Male genitalia of *Roeslerstammia tianpingshana* sp. n., holotype. **A** Dorsum (uncus and tegumen), dorsal view **B** genitalia without phallus, lateral view (basal long hair scales of valva removed) **C** *Ditto* ventral view **D** Right valva, inner view **E** Phallus, lateral view **F** *Ditto*, dorsal view. Scale bar 0.5 mm.

**Figure 9.**
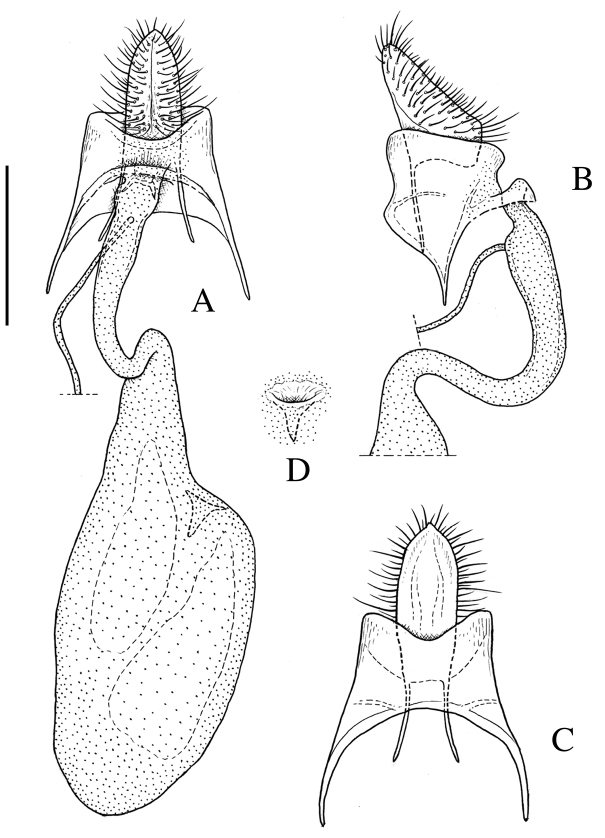
Female genitalia of *Roeslerstammia tianpingshana* sp. n., paratype. **A** Terminalia and bursa copulatrix, ventral view **B** Terminalia, lateral view **C** *Ditto*, dorsal view **D** Signum. Scale bar 0.5 mm.

Figs 4 , 5 , 6 , 7 , 8 , 9

#### Type material.

Holotype male, “Tianpingshan (1,500 m)/ Badagongshan/ Hunan, China/ 26–27.v.2009/ G.H. Huang & M. Li”, in HUNAU. Paratypes 3 males, same label as holotype, in HUNAU; 1 female, same label as holotype, in ELKU.

#### Diagnosis.

Distinguished from other *Roeslerstammia* species by the triangular white marking on the costa of the forewing. In the male genitalia, the uncus is triangular, apically narrow and bilobed; the valva has a blunt process on the median part of the costa; the phallus is long, its basal half straight, distal half strongly upcurved and sinuate. In the female genitalia, the ductus bursae is stout, the caudal part upcurved; the corpus bursae is ellipsoidal, with a thorn-shaped signum.

#### Description.

***Male*** (Figs 4A, 5D).

Forewing length 7.3 mm in holotype, 6.7–7.1 mm in paratypes.

Wing expanse 15.3 mm in holotype, 13.5–14.3 mm in paratypes.

*Head* vertex, including between antennae, with raised blackish brown hairs anteriorly, yellow hairs posteriorly; frons smooth, ochreous with golden luster, laterally along eyes blackish brown with a metallic blue lustre (Fig. 5D). Eyes relatively large, interocular index *ca* 1.0. Antenna filiform, 0.8 >(apical part lost)× as long as forewing; scales in flagellar segments near the middle of antenna somewhat raised (Fig. 7B); scape blackish brown with metallic blue lustre; flagellum dark brown on basal 1/3, white on distal 2/3 (apical part lost). Labial palpus slightly upcurved, relatively long *ca* 2.3 × as long as horizontal eye diameter, 3^rd^ segment slightly longer than 2^nd^; entirely smooth and terminally acute, pale yellow (Fig. 5D).

*Thorax* tegula dark brown with metallic blue luster; mesonotum dark brown with metallic blue or golden luster. Foreleg dark brown, partly mixed with pale yellow; midleg pale yellow with dark brown tibial spurs; hindleg pale browish gray dorsally, tibia with pale yellow hairs ventrally. Forewing lanceolate, apex narrowly rounded, dark brown with metallic blue or golden luster; a clear triangular creamy white marking present at basal 2/3 of costa; fringe dark brown; veins R4 and R5 stalked, R5 reaching to costa (Fig. 6). Hindwing dark brown, darker near apex; fringe dark brown. Hindwing with frenulum consisting of a long bristle.

*Abdomen* dorsal part pale brown with golden luster, terminally with pale yellow tufts of long hairs. Ventral part similar to dorsal part, terminally with creamy yellow smooth scales.

*Male genitalia* (Fig. 8). Uncus triangular, apically narrow and bilobed. Tegumen broad, as long as uncus. Gnathos consisting of two slender arms united medially with a circular plate. Valva broad basally and narrowed distally, with a blunt process on median part of costa; sacculus short, about 1/3 length of valva, terminating in an indistinct projection; a small pad of long hair scales near the base ventrally. Vinculum narrow ventrally; saccus cylindrical, as long as dorsal part of tegumen. Phallus long, basal half straight, distal half upcurved and sinuate, with a band of minute spine-like cornuti.

***Female*** (Fig. 4B).

Forewing length 7.6 mm. Wing expanse 15.8 mm.

Similar to male but differs as follows: antenna filiform, 1.1× as long as forewing. Scales on antennal flagellar segments not raised; flagellum dark brown on basal half and apical 1/7, white on distal half to near apex. Hindwing with frenulum of two slender bristles.

*Female genitalia* (Fig. 9). Papillae anales narrow and apically pointed in ventral view, nearly triangular in lateral view. Apophysis posterioris slender, 0.7 × as long as papilla analis. Apophysis anterioris short and basally broad, 0.3 × as long as eighth tergite. Eighth tergite strongly sclerotized, dorsal posterior margin weakly emarginate at middle. Ostium bursae situated on anterior margin of eighth abdominal segment, posterior margin nearly straight. Ductus bursae stout, caudal part upcurved. Ductus seminalis attached to ductus bursae near ostium. Corpus bursae ellipsoidal, with a thorn-shaped signum.

#### Etymology.

The specific name, an adjective, is derived from the type locality.

#### Host plant.

Unknown. The larvae of *Roeslerstammia* are first leafminers and then become skeletonizers and mainly feed on plants of Betulaceae (*Alnus*, *Betula*) and Malvaceae (*Tilia*) (e.g., Kyrki 1983, Heppner 2005). Recently Hirowatari et al. (2012) recorded Facaceae (*Fagus*) as the hostplant. The type locality of *R. tianpingshana* is located in a deciduous forest where *Fagus lucida* Rehder & Wilson is the dominant tree species. Although we carried out a survey for immature stages of the new species, no information on the hostplant was obtained.

#### Distribution.

China (Hunan Province).

#### Remarks.

On 26–27 May 2009, a total of four males and one female of the new species were collected in a light trap at Tianpingshan (1,500 m), Badagongshan National Nature Reserve, Hunan Province, China. On 12–14 August 2014, we ran a light trap in the same locality, but did not obtain additional material. The adults of *R. pronubella* and *R. erxlebella* are known to fly in spring and summer (Agassiz 1996, Huemer and Segerer 2001, Hirowatari et al. 2012) but we could not confirm the voltinism of the new species.

## Discussion

In the present study, it is confirmed that the new species has all of the following diagnostic characters of Roeslerstammiidae Bruand, [1851] (= Amphitheridae Meyrick, 1913) proposed by Kyrki (1983): forewing R5 reaches to costa, the tegumen is bilobed, the gnathos is present, the valva has a small pad of long hair scales on the outside near the base, the phallus (= aedeagus) is a slender usually curved tube.

Although Heppner (2005) insisted on the validity of Amphitheridae, Nieukerken and Karsholt (2006) concluded that when the type genera of Amphitheridae and Roeslerstammiidae are considered to belong to the same family, the senior name Roeslerstammiidae is the valid family-group name. They regarded that Roeslerstamiidae should be a justified emendationof the originally proposed name Röslertammidae, an incorrect original spelling (following article 32.5.3 of ICZN 1999). In some species of the Roeslerstammiidae *sensu lato*, such as *Telethera* Meyrick, the compound eyes are divided into two parts by scales, extending from the posterior margin (Fig. 7E) (Moriuti 1978, 1987). In the new species (Fig. 7A), the compound eyes are of normal type, as seen in the other *Roeslerstammia* species such as *R. pronubella* (Fig. 7C). Kyrki (1983) pointed out in the redescription of *R. erxlebella* that the scales on the flagellar segments near the middle of the male antenna are somewhat raised, giving them a serrated appearance. This condition may be one of the synapomorphies of *Roeslerstammia* because it is also distinct in *R. tianpingshana* (Fig. 7B) and *R. metaplastica* (=*R. hemiadelpha*).

Among species of *Roeslerstammia*, *R. tianpingshana* is unique in having a blunt process on the median part of the costa of the valva in the male genitalia. In the other species, there is no such process on the costa, but instead a small process is present on the ventral part of the valva. Moriuti (1972) and Kyrki (1983) regarded the small spine-like process present in *R. pronubella* and *R. erxlebella* as the terminal part of the sacculus. In *R. metaplastica*, a blunt process is present on the sacculus, while in *R. tianpingshana*, it is represented by an indistinct projection. If these processes are homologous, the presence of them may be one of the synapomorphies of the genus. In addition, *R. pronubella* and *R. erxlebella* are considered to be closely related in having a protruding sacculus which is approximately 2/3 the length of the valva. In *R. tianpingshana*, the shape and size of the male phallus and female ductus bursae (Figs 8 and 9) correspond well. In the other *Roeslerstammia* species, the shapes of the male phallus are variable and species-specific, but their shapes indicate no clear relationship to the female ductus bursae.

In Japan, *R. nitidella* and *R. bella* were described by Moriuti (1972) focusing on the presence or absence of yellow markings on the hindwing, but they are treated as synonyms of *R. pronubella* and *R. erxlebella*, respectively. Currently it is known that the yellow marking on the hindwings in these species is variable and they are separable by genital characters only (Agassiz 1996, Huemer and Segerer 2001, Hirowatari et al. 2012). As for the Indian species, in the original description of *R. metaplastica*, Meyrick (1921) noted that an elongate spot on the costa is variable in development. Nevertheless, he pointed out many differences in coloration and wing markings between *R. metaplastica* and *R. hemiadelpha*. Thus, the coloration and wing markings of *Roeslerstammia* species tend to be variable and external differences between the two taxa described by Meyrick are attributable to the individual variation or seasonal forms of the same species.

### Check list

A check list of the genus *Roeslerstammia* is provided, based on Heppner (2005). Heppner (2005) treated Japanese representatives of both *R. pronubella* and *R. erxlebella* as distinct subspecies, *R. pronubella nitidella* Moriuti and *R. erxlebella bella* Moriuti. However, as Hirowatari et al. (2012) noted, there is no unique feature in Japanese specimens either in the wing markings or in the genitalia, and thus Moriuti’s species are treated as synonyms.

ROESLERSTAMMIA Zeller, 1839

*erxlebella* (Fabricius, 1787) (*Alucita*) Denmark

*fuscocuprella* (Haworth, 1828) (*Tinea*) England

*chrysitella* (Treitschke, 1833) (*Oecophora*) [Austria?]

*aeneella* (Duponchel, [1839]) (*Adela*) France

*erxlebeniella* Zeller, 1839, emend.

*bella* Moriuti, 1972 Japan

*durulguensis* Budashkin & Kostjuk, 1993 Russia

*pronubella* ([Denis & Schiffermüller], 1775) (*Tinea*) Austria

*transcaucasica* Toll, 1958 Georgia

*nitidella* Moriuti, 1972 Japan

*metaplastica* Meyrick, 1921 India

*hemidelpha* Meyrick, 1922, **syn. n.** India

*tianpingshana* **sp. n.** China

## Supplementary Material

XML Treatment for Roeslerstammia metaplastica

XML Treatment for Roeslerstammia tianpingshana
